# The clinical significance of the human vomeronasal organ

**DOI:** 10.1007/s00276-023-03101-2

**Published:** 2023-02-09

**Authors:** Tjasse D. Bruintjes, Ronald L. A. W. Bleys

**Affiliations:** 1 Department of Otorhinolaryngology, Leiden University Medical Center, Leiden, The Netherlands; 2 Department of Otorhinolaryngology, Gelre Hospital Apeldoorn, Apeldoorn, The Netherlands; 3 Department of Anatomy, University Medical Center Utrecht, Utrecht, The Netherlands

**Keywords:** Septal mucosal pit, Vomeronasal organ, Jacobson’s organ, Septoplasty

## Abstract

**Objective:**

To find out whether the vomeronasal organ (VNO) can be identified in the nose as a mucosal pit in the anterior nasal septum, to elucidate its function in man and to determine whether it is important to preserve the VNO during septal surgery.

**Methods:**

Literature review.

**Results and conclusion:**

The VNO is histologically present in almost all humans, but a macroscopically visible septal pit does not necessarily correspond with the actual VNO. The human VNO is probably a vestigial organ with a non-operational sensory function. It is not necessary to take particular care not to damage the VNO during septal surgery.

## Introduction

**Fig. 1:**
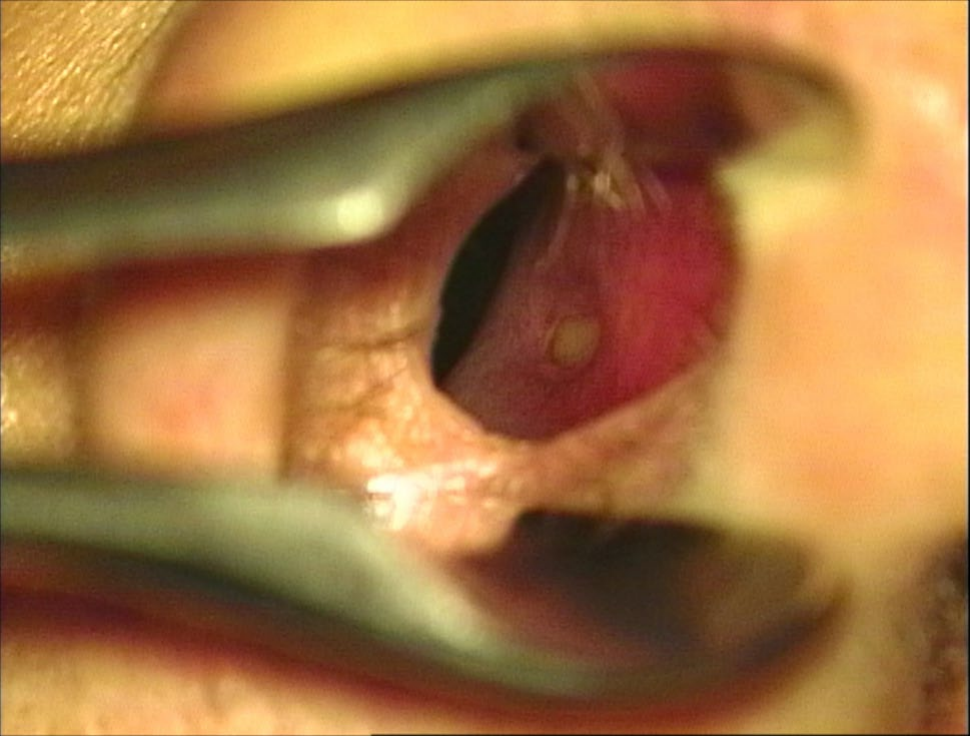
Anterior rhinoscopy (with microscope) showing a clearly visible septal pit on the right side of the anterior nasal septum

In the human nose, a septal pit may be identifiable with the naked eye as a depression in the antero-inferior region of the nasal septum, approximately 2 cm from the nostril (Fig. 1). It may be present on one or both sides of the nasal septum. A septal mucosal pit is actually a common finding, although studies regarding its presence show variable results [7, 9, 12, 14, 15]. Apart from the difference in occurrence rate, the size and shape of the septal pit exhibit considerable variability.

We were curious to know whether such a septal pit corresponds to the opening of the vomeronasal organ (VNO or Jacobson’s organ). The VNO is an accessory olfactory sense organ inside the nose of most amphibia, reptiles and mammals which detects specific chemical compounds, including pheromones [8]. It helps in the process of communicating chemical messages, such as readiness for sexual activity, it helps snakes hunt and track prey, it may also be involved in detecting chemical signals related to aggression. There has been a long-standing debate on the presence and functionality of the VNO in adult humans. If the VNO is a functional organ in humans it would be important to preserve the organ during nasal surgery [5, 6]. In fact, damage may occur quite easily in the presence of a mucosal tear or electrocoagulation of the mucosa of the septum to control bleeding. Foltan & Sedy went as far as to hypothesize that damage to the VNO during orthognatic surgery may lead to VNO function loss affecting the patient’s social life in terms of selecting mates and relations [4].

The aim of our literature study was to find out whether the vomeronasal organ can be identified in the nose as a septal mucosal pit, to elucidate the function of the VNO in man, and to find out whether it is important to take care not to damage the VNO during septoplasty.

## History

The first depiction of the VNO dates from 1703 [2]. The Dutch anatomist Frederik Ruysch was the first to notice the structure in the nose of a cadaver of a 2-year-old boy. However, Ruysch did not give a name to the organ. More than a century later the Danish anatomist Ludwig Jacobson described the organ in detail in some mammals. He was unable to find it in humans and therefore speculated about the VNO being atrophic in humans. Kölliker, in the late nineteenth century, detailed the position of the vomeronasal organ in human embryos as well as adults. In fact, he was the first to provide a histological description of the VNO, he coined the term ‘Jacobson’s organ’ to the VNO and noted the homology with the VNO in other animals. He also described and named the small cartilages below the VNO as Jacobson’s (or paraseptal) cartilages. Bhatnagar and Smith published an overview of the history of the VNO with an English translation of Kölliker’s detailed monograph from 1877 [2].

## Description

A septal pit may contain the opening leading to the VNO, an epithelial, tubular-shaped invagination of the septal mucosa. However, Bhatnagar and Smith observed that the true opening to the VNO is usually microscopic and that a grossly visible septal pit does not always correspond with the actual VNO, but more likely corresponds with the nasopalatine fossa or recess. These structures are related to the incisive canal and located just above the nasal floor, inferior to the opening of the VNO. Bhatnagar and Smith state that more superiorly observed septal mucosal pits may also be unrelated to the opening of the VNO and that only serial section histology can positively identify the true VNO and its opening [1, 3, 13].

**Fig. 2:**
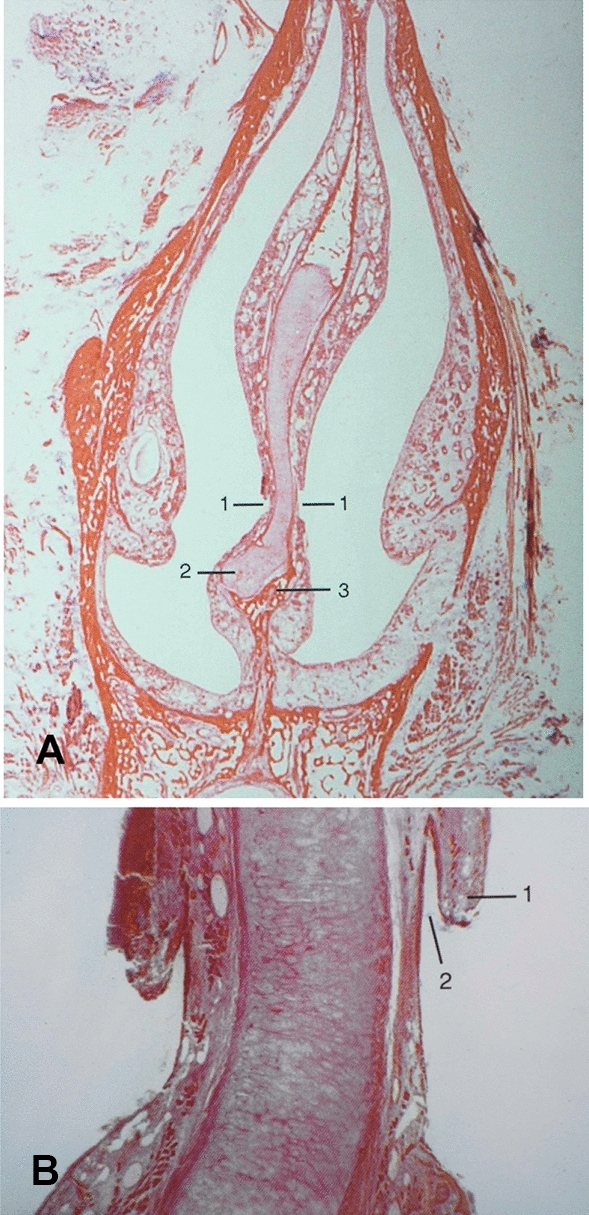
**A** Coronal section of the nose (Mallory-Cason staining, × 2) showing bilateral septal mucosal pit (1), cartilaginous septum (2), premaxilla (3). **B** High magnification detail of **A**, showing the opening of the VNO with posterior lip (1) and beginning of the vomeronasal duct (2)

**Fig. 3:**
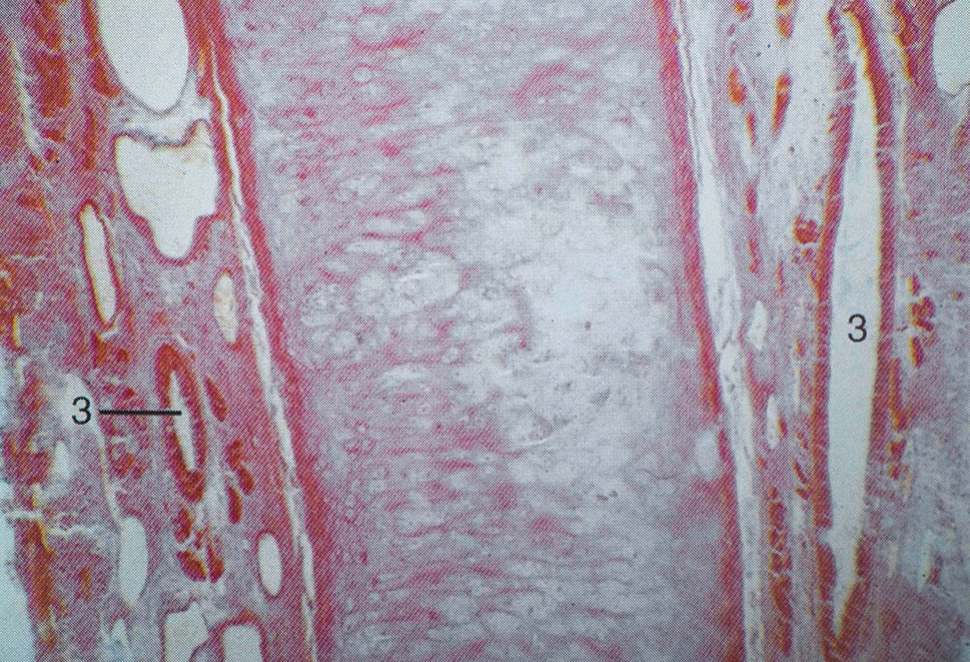
High magnification of nasal septum, showing the VNO (3) lined with a pseudostratified columnar epithelium within the perichondrium of the septal cartilage. Figures 2 and 3 are from the Dept. of Anatomy, University Medical Centre Utrecht, and reprinted from: Huizing, EH, De Groot, JAM. Functional Reconstructive Nasal Surgery. 2nd ed. Stuttgart: Thieme, 2015

Histologic studies reveal that the VNO is present in most, if not all, adult humans [1, 3, 7, 12–14]. It is a small duct of variable length (2–10 mm) underneath the septal mucosa within the perichondrium, which terminates posteriorly in a small pouch. Both duct and pouch are covered with a pseudostratified columnar epithelium with numerous glands (Figs. 2, 3). Whilst neurons are present in the fetal human VNO various studies have failed to identify neurons or nerve bundles in the adult human VNO [7, 17]. In accordance with light microscope observations, electron microscopy studies report three cell types, including basal, and dark and light columnar cells displaying microvilli on the apical surface [12, 14]. The VNO epithelium in humans is quite different from that of the VNO in other species and from that of olfactory or respiratory epithelium in humans [12, 14]. In two histologic studies, the VNO was removed during septoplasty after having received informed consent. Unfortunately, in both studies the patients were not asked about mood or behavioral changes after the procedure [6, 12].

## Function

Whether the vomeronasal organ plays a part in receiving olfactory signals in man has been much debated [10, 16]. The main claim for the functionality of the human VNO comes from the work of Monti-Bloch and colleagues [11]. In humans, they recorded an electrophysiological response after administering putative human pheromones to the VNO region, whilst there was no response when these chemicals were placed in the nasal respiratory mucosa, indicating a selectivity of the VNO. The main arguments against the functionality of the VNO in humans come from anatomical and molecular genetic studies [10, 16]. First and foremost, the VNO in the adult human lacks neurons and nerve fibers. Second, humans are devoid of an accessory olfactory bulb that receives information from the vomeronasal receptor cells, and third, the genes coding for vomeronasal receptor proteins and the specific ionic channels involved in the transduction process identified in species with a functional VNO have mutated and are non-functional. It appears that although the vomeronasal organ is found in the vast majority of human adults, it probably has no function in chemoreception. This does not rule out pheromonal communication in humans, because other sensory systems, such as the olfactory organ, may be involved in pheromone detection. Remarkable pheromone-triggered phenomena in humans, such as the synchronization of the menstrual cycle in women living together, probably follow the main olfactory pathway [16]. Recently, an endocrine function of the VNO has been suggested. The presence of morphological connections of the VNO cells with the underlying capillaries, along with the expression of calcium-binding protein in part of these cells, may indicate endocrine activity [17]. Bhatnagar and Smith suggest that the human VNO functions as a duct for anterior nasal septal glands [1].

## Conclusion

Although the VNO is histologically present in almost all humans, a macroscopically visible septal pit does not necessarily correspond with the actual VNO. The human VNO is probably a vestigial organ with a non-operational sensory function. It is not necessary to take particular care not to damage the VNO during septal surgery.

## Data Availability

Not applicable.
